# Fluralaner, a novel isoxazoline, prevents flea (*Ctenocephalides felis*) reproduction *in vitro* and in a simulated home environment

**DOI:** 10.1186/1756-3305-7-275

**Published:** 2014-06-19

**Authors:** Heike Williams, David R Young, Tariq Qureshi, Hartmut Zoller, Anja R Heckeroth

**Affiliations:** 1 MSD Animal Health Innovation GmbH, Research Antiparasitics, Zur Propstei, 55270 Schwabenheim, Germany; 2 David R Young, Young Veterinary Research Services, 7243 East Avenue, Turlock, CA 95380, USA; 3 Tariq Qureshi, 490 Franklin Circle, Yardley, PA 19067, USA

**Keywords:** *Ctenocephalides felis*, Fluralaner, Flea efficacy, Insecticidal, Reproduction, Dog, Simulated home environment

## Abstract

**Background:**

Fluralaner, a novel isoxazoline, has both acaricidal and insecticidal activity through potent blockage of GABA- and L-glutamate-gated chloride channels. This study investigated the *in vitro* and *in vivo* effects of fluralaner exposure on flea (*Ctenocephalides felis*) reproduction.

**Methods:**

Blood spiked with sub-insecticidal fluralaner concentrations (between 0.09 and 50.0 ng/mL) was fed to fleas for 10 days using a membrane system. Cessation of reproduction in exposed fleas was assessed using flea survival, egg hatchability, and control of oviposition, pupae, and flea emergence. Fluralaner efficacy for *in vivo Ctenocephalides* (*C.*) *felis* control on dogs was assessed using a simulated flea-infested home environment. During a pre-treatment period, dogs were infested twice on days -28 and -21 with 100 adult unfed fleas to establish a thriving population by day 0 of the study. On day 0, one group of dogs was treated with fluralaner (Bravecto™; n = 10), while another group served as negative control (n = 10). Following treatment, dogs were infested three times with 50 fleas on days 22, 50 and 78 to simulate new infestations. Live flea counts were conducted weekly on all dogs for 12 weeks starting 1 day before treatment.

**Results:**

Fluralaner potently inhibited flea reproduction capacity *in vitro*. Oviposition ceased completely at concentrations as low as 25.0 ng/mL. While no ovicidal effect was observed, fluralaner exerted a larvicidal effect at exceptionally low concentrations (6.25 ng/mL). In the simulated flea-infested home environment, flea-control efficacy on fluralaner-treated dogs was >99% at every time point measured for 12 weeks. No adverse events were observed in fluralaner-treated dogs.

**Conclusions:**

Fluralaner completely controls egg laying, larval development and flea reproduction even at sub-insecticidal concentrations. Oral treatment of dogs with fluralaner is highly effective for eliminating fleas in a simulated flea-infested home environment.

## Background

Fluralaner is a new molecular entity of the isoxazoline class that has shown potent acaricidal and insecticidal activity through a dual mechanism of binding to neuronal GABA- and glutamate-gated chloride channels in susceptible invertebrates [1,2]. Fluralaner has high selectivity for arthropods and a very favorable safety profile in vertebrates including dogs [3]. Oral fluralaner administration (Bravecto™) provides 12 weeks efficacy against tick and flea infestations on dogs [4,5].

Flea-adulticide activity on infested dogs is important, but represents only part of the flea-control program needed to effectively eliminate the flea population. The adult population on the dog represents only approximately 5% of the total flea infestation in a dwelling, while the other 95% of the population consists of eggs, larvae and pupae in the dog’s home environment [6]. These maturing stages will re-infest the dog as they become adults.

Effective flea control needs to include highly potent adulticide activity that kills fleas quickly after treatment for immediate relief; maintenance of this high adulticide efficacy through the treatment period; and control of flea reproduction [7]. Fluralaner is a systemic insecticide that kills fleas that feed on treated dogs. The efficacy demonstrated in field trials for both flea control and reduction in signs of allergic flea dermatitis, suggests that treated dogs are exposed to fewer fleas developing from the environmental population of juvenile fleas [5]. Therefore, the objective of the *in vitro* study was to investigate if fluralaner concentrations below the instant flea-killing effect were able to inhibit flea reproduction and thus contribute to the control of environmental flea life stages.

Additionally, a simulated home-environment study was conducted to prove fluralaner’s flea-control properties not only on the dog but also the external flea population that would naturally occur in a household containing an infested animal.

## Methods

### *In vitro* membrane-feeding exposure

A membrane-feeding method [8] was modified to assess the impact of fluralaner exposure on flea reproduction. Defibrinated sheep blood was prepared in a series of dilutions with fluralaner to obtain concentrations between 50.0 and 0.09 ng/mL. Test solutions were prepared twice and each preparation was tested in duplicate resulting in a total of 4 replicates per concentration, along with a fluralaner-negative solvent control (a solvent concentration equivalent to that of the highest concentrated fluralaner test solution) and an untreated control.

Unfed adult fleas (*C. felis;* 20 males and 20 females) were placed into a plastic unit that was then closed with a gauze lid. A grid inside the plastic unit divided the unit into 2 chambers, an upper chamber for flea feeding and a lower chamber for egg collection [8]. Test or control-blood preparations (2 mL) were placed in an artificial membrane-closed glass tube that was then placed on the plastic unit as the food source. Feeding units were incubated (38°C and 60% RH) for 10 days. Test and negative-control-blood preparations were freshly prepared and exchanged (on days 1, 3, 5, and 8) to permit continuous flea feeding. Fleas were transferred into fresh plastic units on days 5 and 8 to facilitate egg collection. Collected eggs were mixed with flea nourishment medium and incubated (28°C and 80% RH) in darkness for 22 (±3) days to enable flea development. Parameters recorded were flea survival, oviposition control, egg hatchability, pupa control and flea-emergence control.

### *In vivo* study to assess flea-control efficacy in a simulated home environment

Twenty healthy male and female mixed breed dogs ≥12 weeks old were housed in individual pens. Ten dogs per group were randomly assigned to receive either a fluralaner chewable tablet (Bravecto™) or no treatment. Each pen contained the bottom half of a dog carrier lined with carpet as bedding. Before treatment, each dog was infested twice, (28 and 21 days pre-treatment) with 100 adult, unfed *C. felis* to establish a flea population prior to treatment on each dog. Flea media was added to the carpet four weeks before the treatment date and weekly thereafter for the remainder of the study to encourage development of an active, developing population of juvenile flea stages in each pen. On the treatment day, dogs in the treated group received fluralaner at a dose close to 25 mg/kg body weight by oral administration of one or more flavored chewable tablets. The chewable tablet(s) were administered by placement in the back of the oral cavity over the tongue to initiate swallowing. Dogs in the negative-control group remained untreated.

Flea counts were performed on all dogs 1 day before treatment, 1 day after treatment and then every 7 days until completion of the study 84 days later. All live fleas recovered were held and re-infested on the dog after the comb count was completed. Each dog was also infested with 50 newly emerged unfed adult fleas on days 22, 50 and 78 to simulate natural infestation post-treatment.

### Statistical analysis

The individual dog was the experimental unit and data from each flea-count time point were analyzed separately. Flea-count data were transformed [Y = log_e_(x + 1)] and analyzed by a mixed linear model including treatment as the fixed effect and block as the random effect. Kenward-Rogers adjustment was used to determine the denominator degree of freedom. A two-tailed F-test was used within the mixed linear model for the comparison between treatment groups and statistical significance was declared when *P* ≤ 0.05. SAS version 9.3 was the primary software used for analysis.

Efficacy was calculated using arithmetic and geometric means with Abbott’s formula:

Efficacy (%) = 100 × (M_C_ - M_T_)/M_C,_ where M_C_ was the arithmetic or geometric mean number of total adult live fleas on untreated dogs and M_T_ the arithmetic or geometric mean number of total adult live fleas on treated dogs.

The study was conducted in California, USA in compliance with the Animal Welfare Act as overseen by the United States Department of Agriculture (USDA) and ethical approval was obtained before the start. The study was approved by the Institutional Animal Care and Use Committee (IACUC no. S11453-00).

## Results

### Impact on flea reproduction after *in vitro* membrane-feeding exposure

Feeding exposure to concentrations of 50 ng fluralaner/mL resulted in a flea survival of 78.1% (day 2), 20.0% (day 3), 8.7% (day 4) and 1.2% (day 5). At 25 ng/mL flea survival rates were 90.6% (day 2), 67.5% (day 3), 31.9% (day 4) and 11.3% (day 5). The flea survival rates increased at lower concentrations (Table 1). Concentrations of 50 and 25 ng fluralaner/mL achieved complete control of oviposition (100%), because fleas that survived 4 to 5 days of feeding at these concentrations did not produce any eggs. At lower concentrations of 12.5 and 6.25 ng fluralaner/mL, the oviposition was controlled by 99.6% and 80.6%, respectively (Table 2). Fluralaner did not affect the hatching of larvae, as hatch was observed in almost all flea groups that were able to lay eggs (Table 3). The pupal development was strongly reduced (85.1% at 12.5 ng fluralaner/mL, 88.7% at 6.25 ng fluralaner/mL) indicating that fluralaner exposure has a potent larvicidal effect (Table 4). The same effect continued through to 100% control of adult emergence at 12.5 ng fluralaner/mL (Table 5).

**Table 1:** Flea survival after feeding on blood containing fluralaner at sub-insecticidal concentrations

**Fluralaner (ng/mL)**	**Flea survival (%)**						
	**Exposure day**^**a**^						
	**2**	**3**	**4**	**5**	**8**	**9**	**10**
50.0	78.1	20.0	8.7	1.2	0	0	0
25.0	90.6	67.5	31.9	11.3	0	0	0
12.5	100	100	67.5	38.9	21.7	17.9	12.3
6.25	100	100	97.5	92.8	85.0	73.1	69.7
3.13	100	100	98.7	97.8	83.1	79.5	78.8
1.56	100	100	99.4	99.1	93.9	92.9	90.3
0.78	100	100	100	100	100	100	100
0.39	100	100	100	100	100	100	98.7
0.19	100	100	100	100	100	100	100
0.09	100	100	100	100	100	100	100

^a^No flea counts were performed on exposure days 6 and 7.

**Table 2:** Flea oviposition control after feeding on blood containing fluralaner at sub-insecticidal concentrations

**Fluralaner (ng/mL)**	**Oviposition control (%)**						
	**Exposure day**^**a**^						
	**3**	**4**	**5**	**8**	**9**	**10**	**Mean**^**b**^
50.0	100	100	100	NA	NA	NA	100
25.0	100	100	100	NA	NA	NA	100
12.5	99.6	100	100	98.9	99.1	100	99.6
6.25	82.6	85.9	81.3	79.9	67.5	86.4	80.6
3.13	32.0	35.7	43.1	70.5	59.9	62.0	50.5
1.56	0	0	17.3	49.8	30.1	29.2	21.1
0.78	8.7	3.0	13.8	12.5	0	18.5	9.4
0.39	6.72	22.8	23.2	23.8	0	20.3	16.1
0.19	0	5.1	21.0	15.1	0	13.9	9.2
0.09	0	11.3	10.9	16.7	0.3	8.1	7.9

^a^No egg counts were performed on exposure days 6 and 7.

^b^Arithmetic mean.

NA: not applicable because all fleas were killed (Table 1).

**Table 3:** Flea larvae emergence from eggs of parent fleas fed on blood containing fluralaner at sub-insecticidal concentrations

**Fluralaner (ng/mL)**	**Larval emergence**					
	**Exposure day**^**a**^					
	**3**	**4**	**5**	**8**	**9**	**10**
50.0	NA	NA	NA	NA	NA	NA
25.0	NA	NA	NA	NA	NA	NA
12.5	no	NA	NA	yes	yes	NA
6.25	yes	yes	yes	yes	yes	yes
3.13	yes	yes	yes	yes	yes	yes
1.56	yes	yes	yes	yes	yes	yes
0.78	yes	yes	yes	yes	yes	yes
0.39	yes	yes	yes	yes	yes	yes
0.19	yes	yes	yes	yes	yes	yes
0.09	yes	yes	yes	yes	yes	yes

^a^No assessment of larval emergence was performed on exposure days 6 and 7.

NA: not applicable because fleas were either killed or did not lay eggs (Table 1 and Table 2).

**Table 4:** Pupal development control from eggs of parent fleas fed on blood containing fluralaner at sub-insecticidal concentrations

**Fluralaner (ng/mL)**	**Pupal development control (%)**						
	**Exposure day**^**a**^						
	**3**	**4**	**5**	**8**	**9**	**10**	**Mean**^**b**^
50.0	NA	NA	NA	NA	NA	NA	NA
25.0	NA	NA	NA	NA	NA	NA	NA
12.5	100	NA	NA	55.2	100	NA	85.1
6.25	90.2	92.4	87.3	86.4	89.9	86.0	88.7
3.13	66.3	68.9	61.7	70.3	62.1	57.4	64.5
1.56	35.3	36.3	34.6	35.5	41.6	27.2	35.1
0.78	7.5	11.4	16.7	10.8	11.9	14.5	12.1
0.39	9.8	0	1.5	3.3	6.3	0.8	3.6
0.19	8.8	0.2	6.8	0	2.7	0	3.1
0.09	9.1	4.5	1.4	0	5.9	2.9	4.0

^a^No pupal counts were performed on exposure days 6 and 7.

^b^Arithmetic mean.

NA: not applicable because fleas were either killed or did not lay eggs (Table 1 and Table 2).

**Table 5:** Adult flea emergence control after parent fleas fed on blood containing fluralaner at sub-insectcidal concentrations

**Fluralaner (ng/mL)**	**Adult flea emergence control (%)**						
	**Exposure day**^a^						
	**3**	**4**	**5**	**8**	**9**	**10**	**Mean**^b^
50.0	NA	NA	NA	NA	NA	NA	NA
25.0	NA	NA	NA	NA	NA	NA	NA
12.5	NA	NA	NA	100	NA	NA	100
6.25	29.2	0	0	9.2	30.8	0	11.5
3.13	4.4	11.9	3.2	8.6	7.5	0	5.9
1.56	0	0	0	10.2	0	0	1.7
0.78	3.8	0	0	3.1	1.8	0	1.5
0.39	0	0	0	1.6	5.2	0	1.1
0.19	4.6	0	1.3	0.8	6.7	0	2.2
0.09	1.1	0.5	0	0	0	0	0.3

^a^No adult flea counts were performed on exposure days 6 and 7.

^b^Arithmetic mean.

NA: not applicable because fleas were either killed or did not lay eggs (Table 1, Table 2, and Table 4).

### *In vivo* flea-control efficacy in a simulated home environment

No adverse events were observed in any fluralaner (Bravecto™) treated dog following administration. Mean flea counts (arithmetic/geometric) on untreated-control dogs were 52.3/26.4 fleas before the day of treatment (day -1) and in the range of 5.1/1.8 to 57.1/40.6 fleas following treatment. Mean flea counts (arithmetic/geometric) on fluralaner-treated dogs were 35.0/14.1 fleas before treatment, 0/0 fleas on days 1, 7, 14, 21, 28, 35, 42, 63, 77 and 84, and 0.1/0.1 fleas on days 49, 56, and 70 after treatment. Compared to control, these counts were significantly different (*P* ≤0.021) on all post-treatment count days. Calculated efficacy results were either 100% or very close to 100% at all post-treatment time points (Table 6).

**Table 6:** Flea-control efficacy on treated dogs (25 mg fluranaler/kg body weight) compared with untreated dogs in a simulated home environment

**Day post treatment**	**Mean flea numbers (arithmetic/geometric)**		**Efficacy**^**a**^**(%)**	**P-value**
	**Control group**	**Treated group**		
-1	52.3/26.4	35.0/14.1	N/A	N/A
1	12.8/6.0	0/0	100/100	0.001
7	5.1/1.8	0/0	100/100	0.021
14	7.1/2.7	0/0	100/100	0.012
21	16.5/4.1	0/0	100/100	0.011
28	53.2/24.8	0/0	100/100	0.000
35	44.1/15.7	0/0	100/100	0.000
42	42.6/10.8	0/0	100/100	0.002
49	48.7/20.6	0.1/0.1	99.8/99.7	0.000
56	57.1/40.6	0.1/0.1	99.8/99.8	0.000
63	42.3/25.6	0/0	100/100	0.000
70	30.0/16.2	0.1/0.1	99.7/99.6	0.000
77	21.9/12.3	0/0	100/100	0.000
84	40.7/33.2	0/0	100/100	0.000

^a^Efficacy calculated from arithmetic/geometric mean flea counts.

NA: not applicable.

## Discussion

Fluralaner has potent inhibitory effects on *C. felis* fleas as demonstrated in the aforementioned *in vitro* and *in vivo* experiments. The control of flea reproduction prevents the formation of a flea population within a household. In addition to a field study that showed fluralaner (Bravecto™) to be effective against fleas on dogs for 12 weeks [5], an *in vitro* study was performed to investigate the effect of fluralaner on flea reproduction using sub-insecticidal concentrations.

The fluralaner concentrations tested in this study were sufficiently low that fleas survived for 2 to 10 days. This survival duration allows for flea reproduction, as viable eggs can be produced 24 hours after fleas start feeding. Fluralaner concentrations tested correlated with the effects on the reproduction cycle. 50 to 25 ng fluralaner/mL effectively controlled the oviposition (egg laying), and 12.5 to 6.25 ng/mL largely reduced the pupal development (indicating a strong larvicidal effect). Overall, sub-insecticidal concentrations as low as 12.5 ng fluralaner/mL achieved 100% cessation of flea reproduction, illustrating that fluralaner provides dog owners’ with additional protection against re-infestation of their pets in the home environment.

The potent *in vitro* effects on flea reproduction substantiate the results of the *in vivo* study where dogs were treated with oral fluralaner (Bravecto™) compared to untreated-control dogs in a simulated home environment. The environment was created by allowing the dogs access to a carpeted bedding area and heavy flea-challenges during the month preceding the fluralaner treatment. This resulted in an environment with a thriving flea population including all developmental stages before treatment administration, leading to an increased flea burden on untreated-control dogs throughout the study duration. Thus, fleas were permanently present on control dogs during the study, however, some variability was observed in flea numbers although the study design should have provided a high flea burden on controls at all assessment times. Following treatment, the flea populations were effectively controlled on the fluralaner (Bravecto™) -treated dogs, with efficacy at, or near, 100% throughout the 12-week post-treatment period.

The long-lasting adulticidal activity of fluralaner (Bravecto™) provides two benefits with respect to flea control: first, it prevents a sustainable re-infestation of dogs by offspring from the environment, and second, it prevents new flea eggs from being added to the environment as female fleas are killed within 8 hours (before they produce eggs) [9]. Both effects lead to the depletion of the environmental flea population. The studies described here indicate that fluralaner’s flea-control capabilities combine an adulticidal effect with long-term efficacy and, additionally, effective prevention of flea reproduction. This is an advantage over pure adulticides which are often combined with an insect-growth regulator (IGR) to provide the same effect.

Highly effective control of environmental flea populations has been recorded with topically applied insecticides [7], but is not reliably achieved with previously evaluated systemically administered insecticides [10]. Taken together the results of both the *in vitro* and *in vivo* studies support the claims of fluralaner’s effective flea control properties. In addition to the fast flea killing effect within 8 hours [9] the studies indicate that fluralaner, a systemic flea treatment, is successful in controlling developing flea populations in the environment.

The efficacy of monthly treatments is dependent on dog owner compliance. It has recently been shown that owner compliance with monthly re-treatment recommendations is weak [11] which may jeopardize flea control. The results reported here provide evidence that a single systemic fluralaner treatment (Bravecto™) provides 12 weeks of flea-population control and is a valuable new tool for achieving effective and long-term flea control for dogs and their homes.

## Conclusions

Fluralaner is a potent inhibitor of flea reproduction at various developmental stages and at exposure levels that are far below its immediate insecticidal activity. Thus, fluralaner treatment disrupts the flea-breeding cycle and protects dogs and their homes from flea infestations over a 12-week period without additional premise treatment.

## Competing interests

HW, AH and HZ are all employees of Merck/MSD Animal Health. TQ was an employee of Merck/MSD Animal Health during the time the study was conducted in dogs. DRY provided contract research support.

## Authors’ contributions

HW, HZ and AH prepared the *in vitro* study design and protocol. HZ assisted in conducting the *in vitro* study and was responsible for data analysis. DY and TQ prepared the *in vivo* study design and protocol and were responsible for data analysis. HW drafted the manuscript and all authors revised and approved the final version.
